# Seed lost to perineum from hydrogel spacer after brachytherapy for prostate cancer

**DOI:** 10.1002/iju5.12579

**Published:** 2023-03-09

**Authors:** Makoto Nakiri, Kosuke Ueda, Ryuji Hoshino, Naoyuki Ogasawara, Hirofumi Kurose, Kiyoaki Nishihara, Koichiro Muraki, Chikayuki Hattori, Etsuyo Ogo, Tsukasa Igawa

**Affiliations:** ^1^ Department of Urology Kurume University School of Medicine Kurume Japan; ^2^ Department of Radiology Kurume University School of Medicine Kurume Japan

**Keywords:** brachytherapy, hydrogels, inflammation, perineum, prostate cancer

## Abstract

**Introduction:**

We describe a rare case of brachytherapy for prostate cancer in which a seed was lost from the perineum after a hydrogel injection.

**Case presentation:**

A 71‐year‐old Japanese man was diagnosed with localized high‐risk prostate cancer. Trimodality therapy with I‐125 brachytherapy was selected, and combined androgen blockade therapy was initiated. Brachytherapy and hydrogel injection were performed 7 months after combined androgen blockade initiation; 6 months later, the patient visited our hospital with complaints of redness and bleeding in the perineum. Serous effusion and loss of a seed on the right side of the perineal anus were observed. Pelvic magnetic resonance imaging showed a tunnel like discharge of hydrogel from the dorsal prostate to the perineum. The fistula was incised, the seed was removed, and drainage was performed.

**Conclusion:**

Appropriate diagnosis and treatment with careful follow‐up are required in patients at high risk of infection after brachytherapy with hydrogel injection.

**Abbreviations & Acronyms**
BT: brachytherapy
CAB: combined androgen blockade
HbA1c: glycosylated hemoglobin
MRI: magnetic resonance imaging

**Keynote message**

This is the first report of a seed lost to the perineum from a hydrogel spacer after brachytherapy for prostate cancer. Appropriate diagnosis and treatment are important for patients at high risk of infection after brachytherapy with hydrogel spacer injection. Furthermore, careful follow‐up is required in these patients.

## Background

Hydrogel is a gelatinous material mainly composed of polyethylene glycol. It is inserted between the prostate and the rectum to maintain the distance and to reduce the radiation dose to the rectum from radiotherapy.^1^ In this report, we describe a rare case of BT for prostate cancer in which a seed was lost from the perineum after the hydrogel injection.

## Case presentation

**Fig. 1:**
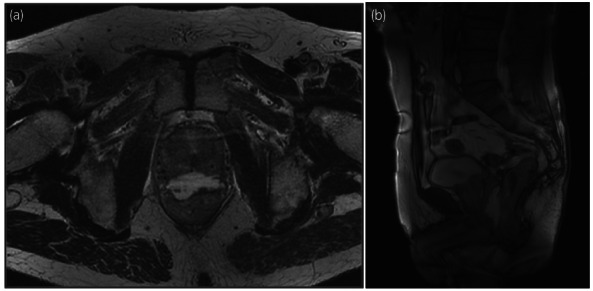
MRI after BT and the hydrogel spacer injection ––horizontal (a) and sagittal (b) views.

**Fig. 2:**
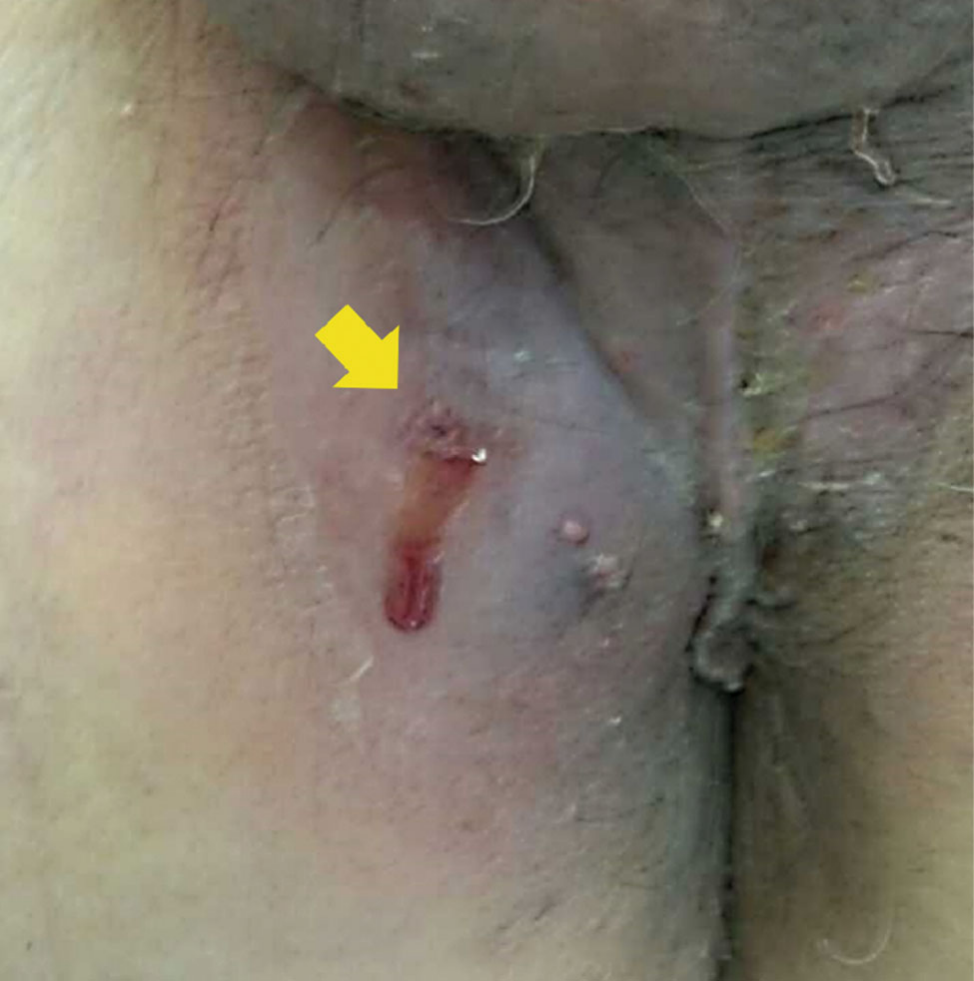
Serous effusion and loss of a hydrogel seed from the fistula on the right side of the perineal anus.

**Fig. 3:**
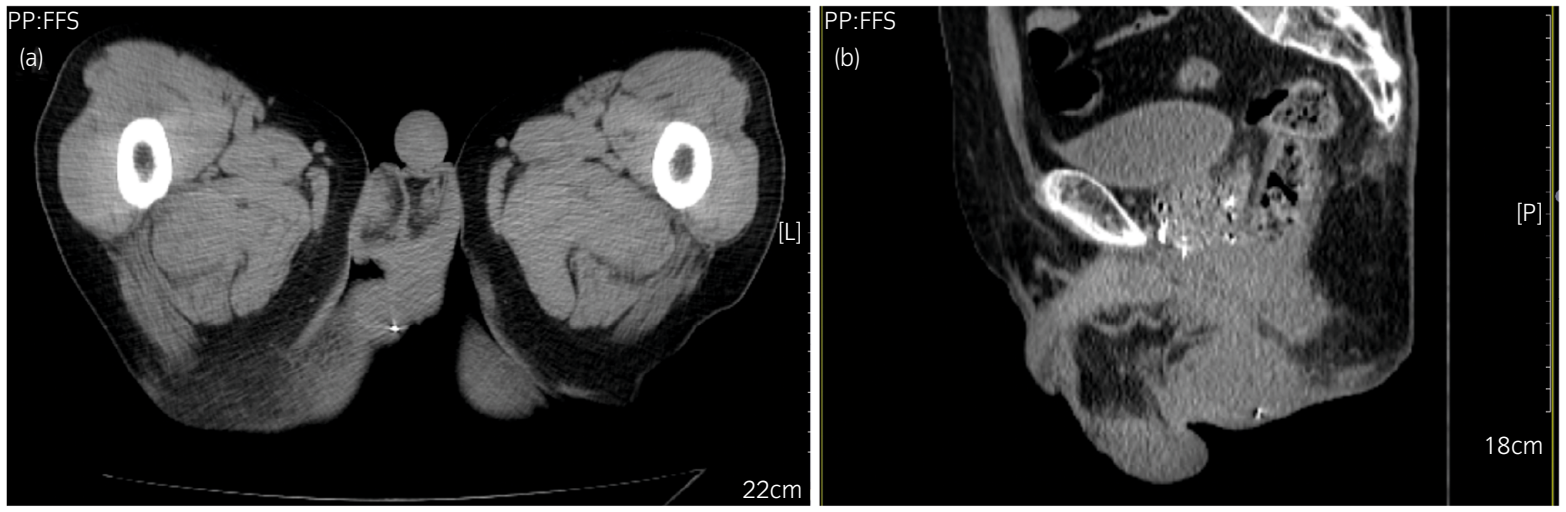
Abdominal computed tomography showed high density area suggestive of a hydrogel seed on the right side of the perineum––horizontal (a) and sagittal (b) views.

**Fig. 4:**
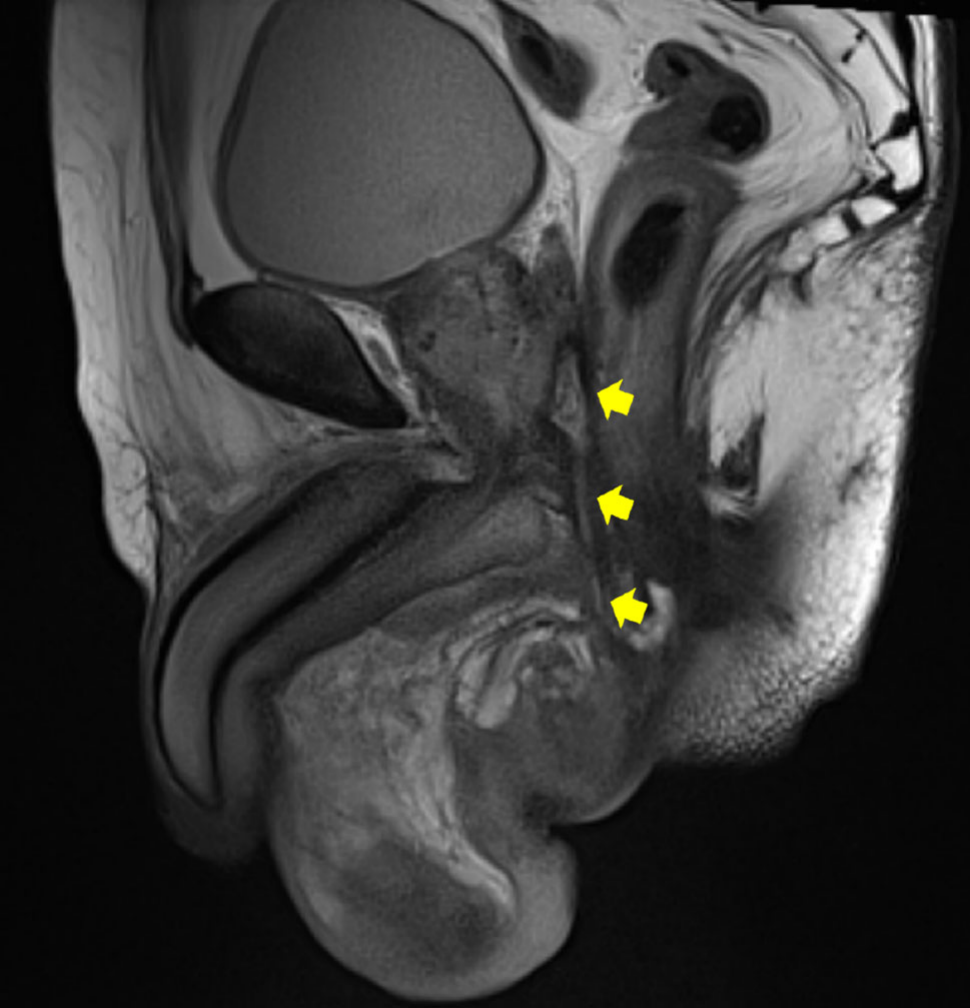
MRI showed tunnel‐like discharge of hydrogel from dorsal prostate to perineum.

A 71‐year‐old Japanese man was diagnosed with localized high‐risk prostate cancer (cT2bN0M0, Gleason Score: 4 + 5 = 9, initial prostate specific antigen: 18.9 ng/mL). Trimodality therapy with I‐125 BT was selected, and CAB therapy with bicalutamide and goserelin was initiated. BT (prescribed dose: 110 Gy) and a hydrogel spacer injection were performed 7 months after CAB initiation (Fig. 1). Seventy seeds were placed during BT without complications or loss. The external beam radiation therapy (prescribed dose: 45 Gy) was performed 1 month after BT. The patient visited our hospital with complaints of redness, pain, and bleeding in the perineum 6 months after BT and the hydrogel spacer injection. We observed erythema, swelling, and induration on the right side of the perineal anus; the formation of a pinhole fistula in the center of the lesion (Fig. 2); and serous effusion with loss of a seed from the fistula. The laboratory tests revealed elevated C‐reactive protein levels to 5.91 mg/dL (normal range, ≤0.14 mg/dL) and elevated blood glucose levels to 230 mg/dL. In addition, his HbA1c was 5.8% before BT and had increased to 7.6%. Abdominal computed tomography showed a high‐density area suggestive of a seed on the right side of the perineum and inflammatory findings in the surrounding tissue (Fig. 3). Pelvic MRI showed a tunnel‐like discharge of hydrogel from the dorsal prostate to the perineum (Fig. 4). The fistula was incised, the seed was removed, and drainage was performed. Both the perineal drainage and urine cultures were negative. The patient received antibiotic therapy and underwent wound cleaning, and the inflammation improved thereafter (based on receding erythema and reduced C‐reactive protein levels). Indigo carmine was injected through the perineal fistula to evaluate for urethral and rectal fistulas, neither of which were observed. After the inflammation subsided, the fistula site was sutured. Two years after treatment, no recurrence of prostate cancer or relapse of inflammation has been observed.

## Discussion

Hydrogel is inserted transperineally with a puncture needle under transrectal ultrasound guidance, and injected between the prostate and rectum, creating an approximately 1 cm distance. Hydrogel maintains its shape for about 3 months before liquefying, being absorbed, and ultimately excreted by the kidneys after approximately 6–12 months. The procedure is easy, with a success rate of 97%.^2^

Miller *et al*. reported that the rectal v70 (volume ratio of the rectum irradiated with ≥70 Gy) was reduced by 66% in the hydrogel placement group. It has been reported that hydrogel injection is associated with reduced acute and late ≥grade 1 serious rectal toxicity and reduced risk of ≥grade 2 serious rectal toxicity by 77% in long‐term follow‐up.^2^ Additionally, Vagger *et al*. reported proctitis, rectal bleeding and ulcers, rectourethral fistulas, and anaphylactic shock as complications associated with hydrogel injection.^3^ There have also been reports of perineal abscesses^4^ and rectal perforation^5^, ^6^ as serious complications; however, seed loss from the perineum has not been previously reported. In this case, retrospective imaging studies led us to believe that a seed placed near the coating on the right side of the dorsal prostatic margin had fallen out of the perineum.

The following three factors were thought to have contributed to this episode. First, the injection of hydrogel caused physical compression, resulting in ischemic changes around the perineum and rectum.^7^ Second, this patient had comorbid type 2 diabetes, with post‐BT HbA1c of 7.8% and poor glycemic control. Hypoxia at the hydrogel injection site and delayed wound healing were induced due to vascular dysfunction and neuropathy.^8^ Third, in order to improve the cancer control rate and reduce adverse events, we placed the seed along the outer capsule of the prostate, which is in contrast with the usual implantation method. As a result, the seed placed on the right side of the dorsal prostatic margin was lost. Radiation‐induced inflammatory changes associated with the lost seed and external irradiation occurred in the perineum. We speculate that the liquefied and eluted hydrogel formed another fistula and reached the perineum through the puncture, engulfing the seed that had been lost near the coating on the dorsal prostatic margin.

It has been reported that MRI is able to clearly visualize hydrogel seeds during post‐injection evaluation.^9^ Another report states that MRI is also useful for evaluating perirectal fistulas after hydrogel injection.^10^ In this case, pelvic MRI was taken as part of the normal post‐planning, 1 month after the implantation, revealing no problems with the hydrogel insertion. In this report, the comparison with the MRI of the perineal fistula significantly contributed to the diagnosis.

Hydrogel injection is generally indicated for prostate cancer patients undergoing radiotherapy. However, due circumspection should be observed for patients with suspected cancer invasion outside the lateral prostate capsule; those with seminal vesicle gland invasion; or those at high risk of infection, such as those with severe diabetes. Since this patient had type 2 diabetes, the indication needed to be carefully considered. However, there were no problems with blood glucose control at the time of injection, so it was decided that the patient was indicated for hydrogel injection. Nevertheless, since hydrogel remains in the body for several months after injection, blood glucose control is considered important before and after surgery.

Various reports have shown adverse events related to hydrogel injection. On the subject of remission with conservative treatment, there are reports of spontaneous remission with low‐fiber diet for rectal ulcer, remission of perineal abscess after administration of antibiotics and drainage^3^; and remission due to administration of antibiotics and hyperbaric oxygen for rectal perforation.^5^ In this case, no rectal ulcer, abscess, or rectourethral fistula was observed, and spontaneous remission was observed following conservative treatment with antibiotics and drainage. However, in patients with delayed wound healing and a high risk of infection, appropriate diagnosis and treatment along with careful follow‐up, are important in the perioperative period and beyond.

## Author contributions

Makoto Nakiri: Conceptualization; data curation; investigation; methodology; project administration; resources; validation; writing – original draft; writing – review and editing. Kosuke Ueda: Project administration; writing – original draft; writing – review and editing. Ryuji Hoshino: Conceptualization; data curation; methodology; project administration; writing – original draft; writing – review and editing. Naoyuki Ogasawara: Data curation. Hirofumi Kurose: Data curation. Kiyoaki Nishihara: Data curation; project administration. Koichiro Muraki: Conceptualization; investigation; resources. Chikayuki Hattori: Methodology; resources. Etsuyo Ogo: Conceptualization; supervision. Tsukasa Igawa: Conceptualization; project administration; supervision; writing – review and editing.

## Conflict of interest

The authors declare no conflict of interest.

## Approval of the research protocol by an Institutional Reviewer Board

Not applicable.

## Informed consent

The patient provided written informed consent for all treatments.

## Registry and the Registration No. of the study/trial

Not applicable.
